# Microcarrier-seeded muscle cells exhibit delayed differentiation in simulated microgravity compared to a terrestrial bioreactor

**DOI:** 10.1038/s41538-025-00498-5

**Published:** 2025-07-25

**Authors:** Hamed Alizadeh Sardroud, Mahdieh Shokhrollahi Barough, Esfandyar Askari, Mohsen Akbari

**Affiliations:** 1 Laboratory for Innovations in Micro Engineering (LiME), Department of Mechanical Engineering, University of Victoria, Victoria, BC Canada; 2 Terasaki Institute for Biomedical Innovations, Los Angeles, CA USA

**Keywords:** Biotechnology, Biomaterials

## Abstract

This study explores the feasibility of microcarrier-seeded muscle cell expansion and differentiation in simulated microgravity (µG) conditions, aiming to develop a proof-of-concept for producing cultivated meat in space. Gelatin microcarriers supported C2C12 expansion and myogenesis in static culture. The microcarriers were cultured in 1G (stirred bioreactor) and simulated µG bioreactors. µG slowed down the cell expansion, while the 1G condition showed a significant increase in cell expansion. Cells exhibited nuclear elongation and extended cell bodies over 7 days in both 1G and µG conditions. Flow cytometry and real-time polymerase chain reaction (RT-PCR) revealed enhanced myogenesis in both 1G and µG, though differentiation was delayed and gene expression significantly lower under µG. These results suggest that while µG initiates differentiation, the process is primarily limited to early stages. Despite the slower myogenesis, it remains feasible, and future research should focus on culture conditions to enhance muscle cell functionality in µG.

## Introduction

The extensive plans of major space agencies, including the National Aeronautics and Space Administration (NASA) and the European Space Agency (ESA), for sustained human habitation in space have spurred missions to the Moon and Mars^1–3^. This ambition is shared by prominent companies such as Virgin Galactic, SpaceX, and Blue Origin, all striving towards similar goals in advancing space exploration^4,5^.

Food supply in space missions is critical for astronaut health and well-being. Spaceships transport fresh fruits and vegetables, as well as freeze-dried or canned foods, for space travel. Additionally, specialized delivery missions can be conducted to supply food from Earth to spaceships in space^6^. Fresh foods have a limited shelf-life, while processed foods lack the diverse nutrients necessary for space travelers, potentially leading to various health issues^7,8^. Canning, drying, and freeze-drying are reliable for safe, long-lasting space foods, but they reduce nutritional value and quality. For long missions, bringing enough food from Earth is impractical, and relying solely on preserved foods can lead to nutritional deficiencies^9,10^. Therefore, food production in fully operational space facilities is the sole option for supplying fresh food to space travelers. Meat is a dietary essential for most individuals due to its high protein availability of around 70%^11^. It offers necessary animal fats, and essential unsaturated fatty acids, along with vital mineral elements like iron, zinc, and phosphorus crucial for bodily functions^12^. Raising farm animals for meat production in space or on other planets is impractical in the near future due to the difficulties of transporting large animals and the need for specialized facilities to house and feed them^13^.

Lab-grown meat has recently advanced significantly, evidenced by the introduction of the first restaurants offering this product in Singapore in 2020^14^. Lab-grown meat is produced by growing animal cells in a bioreactor, offering an alternative to conventional meat sourced from slaughtered animals^14^. As a result, lab-grown meat serves as a feasible alternative to preserved foods for space missions. In 2001, NASA successfully produced lab-grown muscle tissue, sourced from goldfish, with lengths ranging from 3 to 10 cm, in Petri dishes^15^. Currently, various companies, including Aleph Farms, are exploring the possibilities of lab-grown meat for space travel^16^. However, space experiments encounter major obstacles. They require sophisticated equipment to handle extreme conditions, are highly expensive, and involve prolonged processes for setup, operation, data collection, and result verification^17^.

Various devices have been designed to mimic microgravity (µG) conditions on Earth, addressing challenges encountered in space research. Devices such as clinostats, rotating wall vessels (RWV), and random positioning machines (RPM) recreate µG environments in terrestrial laboratories^18^. These cost-effective and easily developed tools provide a solution for examining cell culture phenomena in simulated µG environments, facilitating research relevant to space exploration^19^. The RWV platform mimics µG for cell culture by acting as a specialized clinostat, imparting rotational velocity to cells within a fully fluid-filled vessel, enabling their movement in circular paths^20^. In this system, cells cannot be cultured as monolayers, requiring microcarriers for adherent cell growth^21^. Soft edible microcarriers, in particular, can mitigate or entirely eliminate the need for dissociation, degradation, and separation processes of microcarriers at the end of process^22^. This is particularly beneficial in space µG environments, where these processes are challenging and expensive^23^. Thus, cells can be seeded onto edible microcarriers and cultured in bioreactors. Once differentiation into myotubes and the production of meat extracellular matrix (ECM) on the microcarriers are complete, the edible biomaterial can be incorporated into the final meat product and consumed^22^.

The principal aim of the current study was to establish a proof-of-concept approach and explore the possibilities for muscle cell expansion and differentiation for cultivated meat production, with a focus on applications in space environments. The hypothesis is that µG conditions impair the expansion and differentiation capacity of microcarrier-seeded muscle cells. We will test this by focusing on analyzing cells expansion and different gene expressions to identify differences in muscle cell differentiation stages by performing a binary experimental design, examining a terrestrial 1G bioreactor and a µG -simulating bioreactor. Specifically, the study focuses on markers across the myogenesis pathway, including proliferation/early myogenesis (PAX7, MYOD), mid differentiation (MYOD, MYOG), and late differentiation and maturation (MYH4, CSRP, and MSTN). Gelatin was chosen as a soft edible biomaterial because of its arginylglycylaspartic acid (RGD) sequence, which promotes cell adhesion^24^. We intended to examine how muscle cells seeded on edible microcarriers respond to µG conditions, evaluating cell viability, expansion, and myogenic differentiation in both terrestrial and simulated µG scenarios. The present methodology could be an earth-based analytical tool for investigating cultivated meat for space-related applications.

## Results

### Microfluidic gelatin microcarriers fabrication and optimization

**Fig. 1:**
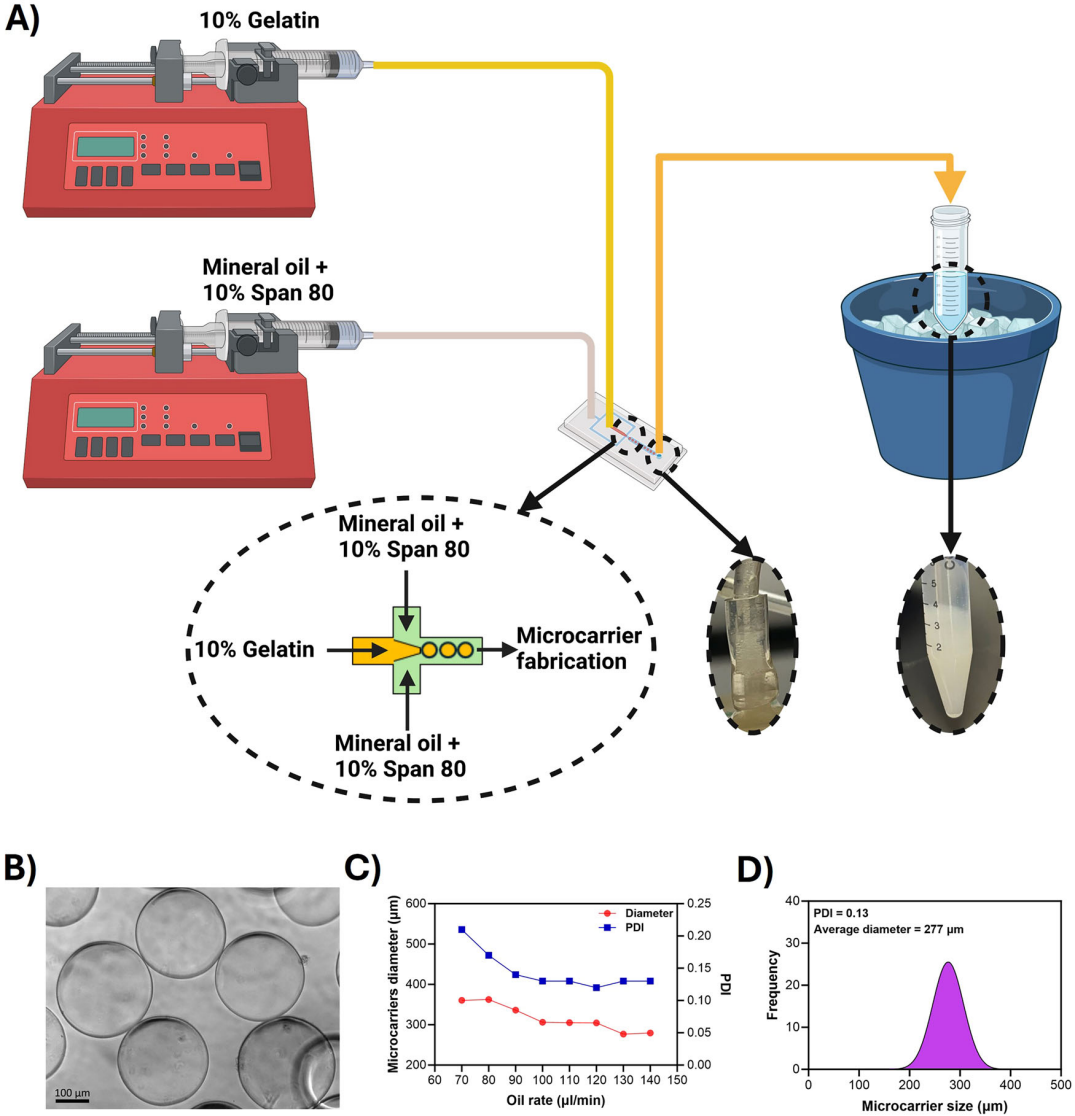
Fabrication and Optimization of Gelatin Microcarriers Using Microfluidics. **A** Gelatin microcarriers were fabricated using a flow-focusing microfluidic device and collected in a 15 ml tube placed in an ice bath. **B** The microcarriers were crosslinked with transglutaminase, resulting in spherical shapes as shown in the image. **C** Increasing the oil flow rate decreased both the PDI and the diameter of the microcarriers (n = 1). **D** An oil flow rate of 130 resulted in a PDI of 0.13 and a mean diameter of 277 µm; this flow rate was chosen for fabricating gelatin microcarriers for cell culture. Statistical analyses were not performed on these measurements as each was repeated only once by increasing the flow speeds from 70 to 140 µl/min. Created with BioRender.com.

In this study, we aimed to establish a proof-of-concept for cultivated meat research in the context of µG applications. To this end, we sought to fabricate edible gelatin microcarriers using previously developed flow-focusing microfluidic devices in our lab, which have been utilized and validated for other research purposes^25,26^ (Fig. 1A). Our goal was to produce microcarriers with diameters of less than 300 µm. In line with this goal, we maintained the gelatin flow rate at a consistent 10 µl/min while increasing the oil rate from 70 to 140 µl/min. The fabricated microcarriers were spherical in shape (Fig. 1B). The diameter and Polydispersity Index (PDI) of the fabricated microcarriers were measured and showed a consistent reduction in microcarrier diameter from 360 ± 76 μm to 277 ± 35 μm, accompanied by a decrease in PDI from 0.21 to 0.13 (Fig. 1C). In this study, we chose to work with the smallest microcarriers (diameter of 277 µm ± 35) and a PDI of 0.13, due to their highest surface to volume ratio (Fig. 1D).

### Differentiation media enhanced the expansion and differentiation of the C2C12 cells

**Fig. 2:**
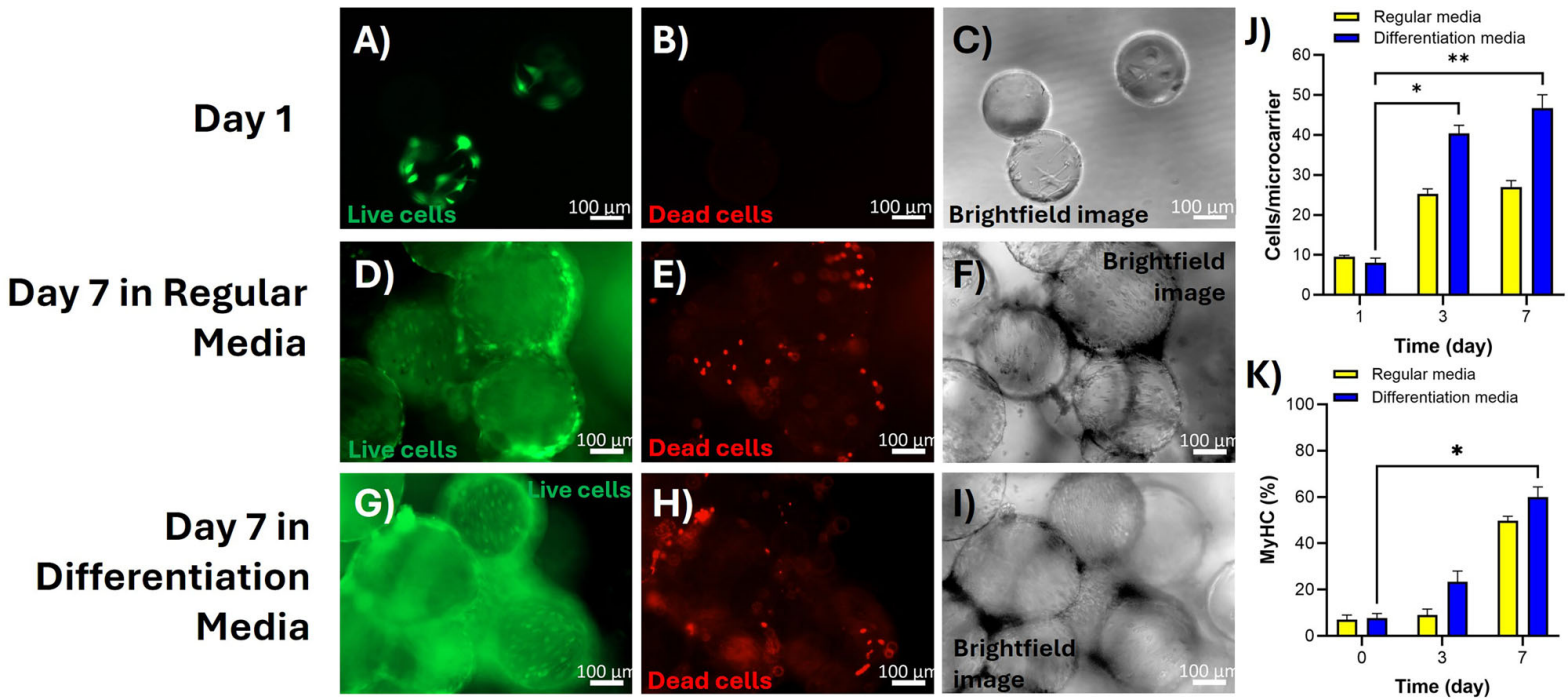
Expansion and differentiation of the C2C12 cells on microcarriers in static culture. **A**, **D**, **G** Calcein-stained live cells (green) on gelatin microcarriers at day 1 and day 7 demonstrated increased cell viability over time in both regular (**D**) and differentiation media (**G**). **B**, **E**, **H** Ethidium homodimer-1-stained dead cells (red) on day 7 showed minimal cell death compared to the population of the live cells. **C**, **F**, **I** Brightfield images depicted microcarriers with attached cells on day 8, contrasting with uncovered microcarriers on day 1. Only the attached cells to the microcarriers were counted after enzymatically detaching them; **J** Quantification of cell expansion per microcarrier revealed higher expansion in differentiation media (*n* = 3). **K** Flow cytometry analysis of MyHC showed significant upregulation in differentiation media (*n* = 3). * and ** indicate significant differences at *p* ≤ 0.05 and *p* ≤ 0.01, respectively.

We first established the effect of differentiation media on cell expansion and differentiation on microcarriers. To this end, C2C12 cells were seeded on gelatin microcarriers and cultured for 7 days in both regular and differentiation media and a static condition. Cell viability and expansion were assessed using trypan blue and live/dead staining. Viable cells were observed on the microcarrier surfaces 1 day after seeding, and no dead cells were observed initially, demonstrating the excellent cytocompatibility of the gelatin microcarriers (Fig. 2A–C). As expected, the cells covered only a few microcarriers on day 1, but by day 7, they covered the surface of all microcarriers in both media types (Fig. 2D, G). According to the live/dead staining, cells expanded and even bridged between microcarriers, indicating that these microcarriers provided sufficient adhesion sites for cells to attach and grow (Fig. 2D, G). Quantitative analysis using trypan blue staining demonstrated a statistically significant increase (*p* = 0.0011) in cell number in differentiation media compared to regular media on day 7, suggesting that differentiation media is more effective for cell expansion (Fig. 2J). Dead cells were observed in both conditions after 7 days of culturing, but their presence was minimal compared to the intense green fluorescence of live cells (Fig. 2E, H).

The cells were labeled with MF-20, an antibody that binds to the myosin heavy chain (MyHC) protein to assess myotube formation. MyHC is predominantly expressed in myotubes, and changes in fluorescence through flow cytometry analysis confirm the presence of myogenesis. Our results indicated a gradual increase in MyHC fluorescent intensity over time in C2C12 cells cultured in both media types (Fig. 2K). The increase in MyHC-positive cells cultured in differentiation media over 7 days was statistically significant (*p* = 0.0412) (Fig. 2K). Based on the results of this preliminary static culture, differentiation media was chosen for the dynamic culture of the cells.

### µG condition inhibited cell expansion

**Fig. 3:**
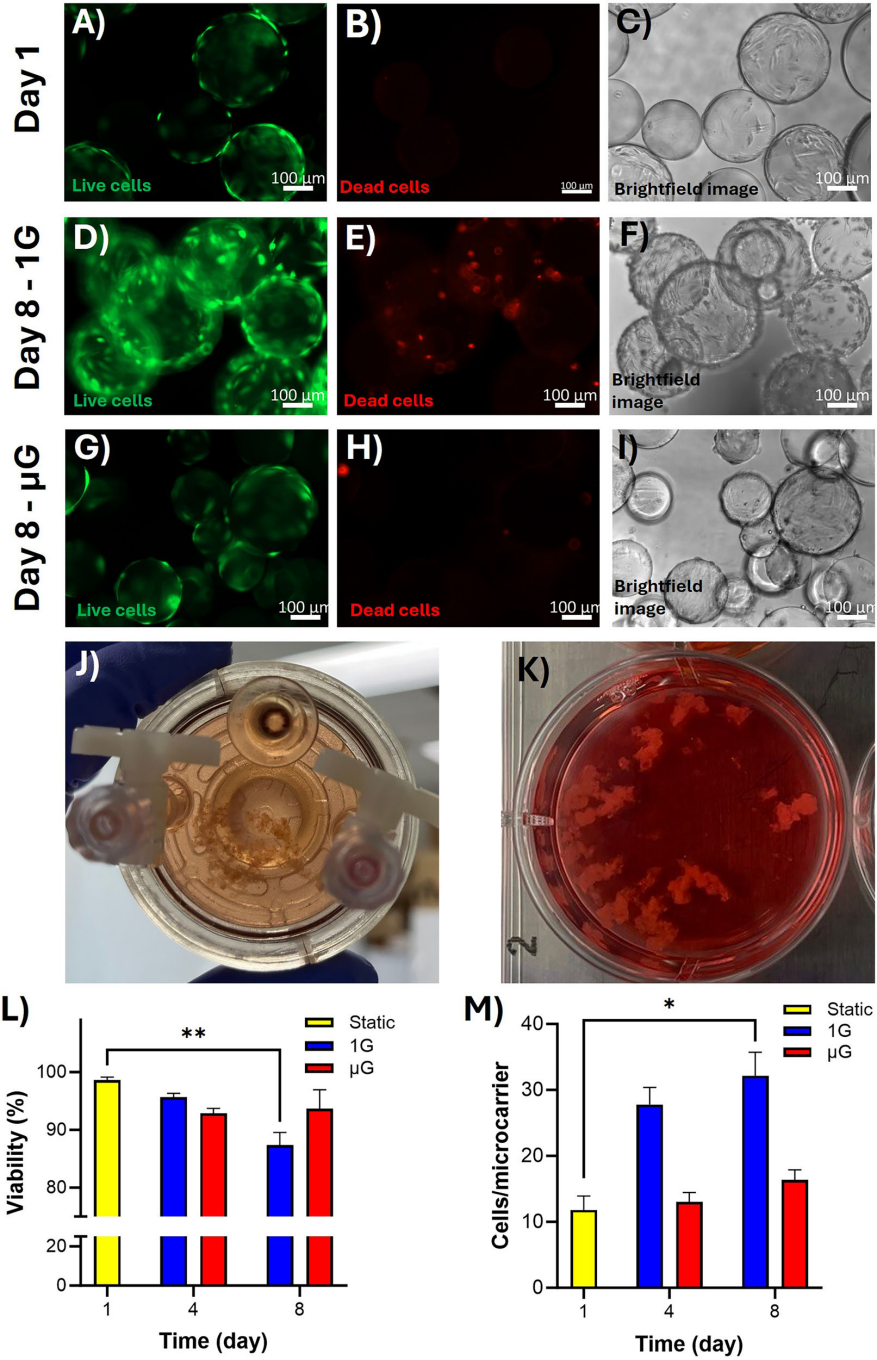
Expansion and differentiation of the C2C12 cells on microcarriers cultured in µG and 1 G bioreactors. **A** Viable cells were observed at day 1 in static culture. **D**, **G** In dynamic conditions, viable cells were observed across all groups, yet green fluorescence intensity was notably higher in microcarriers from the 1G bioreactor compared to those from the µG. **B**, **E**, **H** Presence of dead cells was noticeable in 1G bioreactor condition compared to day 1 and µG condition. **C, F, I** Brightfield images depicted microcarriers with attached cells on day 8. **J**, **K** Images shows that microcarriers created aggregates in both 1G and µG bioreactors, which were visible to the naked eye. Only the attached cells to the microcarriers were counted after enzymatically detaching them; **L** cell viability assessment showed nearly 100% viability on day 1, with a decrease observed in the 1G bioreactor by day 8. **M** Quantification of cell expansion per microcarrier revealed higher cell numbers in the 1G bioreactor compared to the µG condition. *n* = 4 and * and ** indicate significant differences at *p*  *≤* 0.05 and *p*  *≤* 0.01, respectively.

Cell-seeded microcarriers were cultured with differentiation media in two dynamic conditions, including 1G (stirred bioreactor) and µG. The viability of C2C12 cells on the microcarriers was evaluated using live/dead staining. Viable cells were visible on the microcarrier surfaces 1 day after seeding (Fig. 3A). After 7 days of culturing in the bioreactors, viable cells were observed in both 1 G and µG groups (Fig. 3D, G). Notably, green fluorescence intensity appeared higher in microcarriers from the 1G bioreactor compared to the µG condition (Fig. 3D, G). On day 8, there was a noticeable presence of dead cells in the 1G bioreactor, despite signs of increased cell expansion in this condition (Fig. 3E). The formation of microcarrier aggregates was also observed in both the 1G and µG bioreactors (Fig. 3J, K). The aggregation of microcarriers can enhance cell-cell interactions and mimic the natural muscle tissue environment, which is beneficial for cultivated meat production. Trypan blue staining was employed to enumerate live and dead cells during culture in bioreactors. Cell viability was nearly 100% on day 1 of culturing on the microcarriers (Fig. 3L). Cell viability was assessed on days 4 and 8, revealing that both dynamic conditions maintained viability above 92%, except for the 1 G bioreactor on day 8, which decreased to 87.33% (Fig. 3L). This reduction was statistically significant (*p* = 0.0041) compared to the viability observed on day 1. Cell expansion was quantified by counting live cells relative to the number of microcarriers in each group (Fig. 3M). Throughout a 7-day period, there was only a slight increase in cell number per microcarrier in µG condition. In contrast, a statistically significant increase (*p* = 0.026) was observed in the 1G bioreactor (Fig. 3M), as evidenced by increased live cell staining on the microcarriers from day 1 to day 8 (Fig. 3A, D).

### Cells elongated in both 1 G and µG conditions

**Fig. 4:**
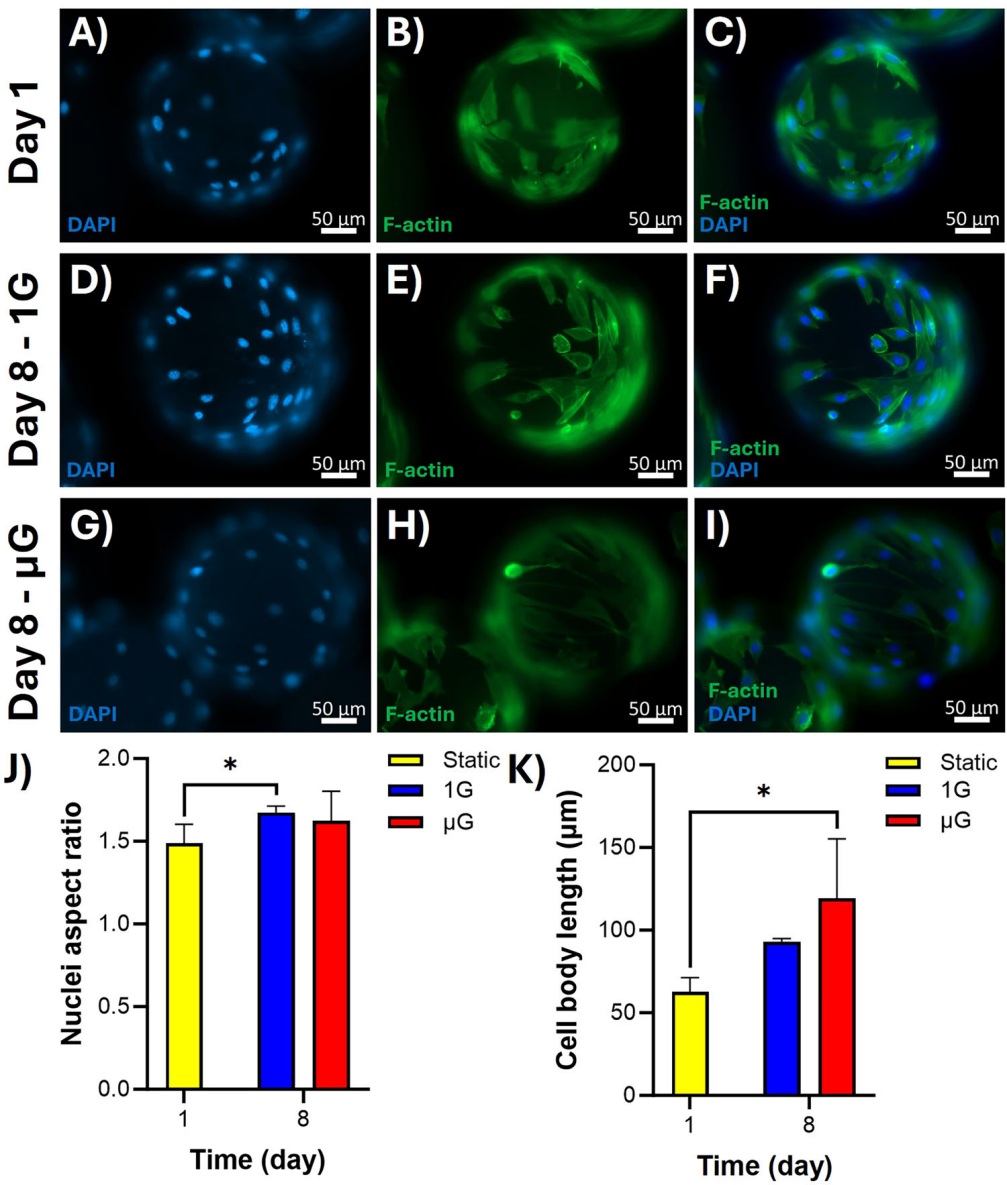
Immunofluorescence staining of nuclei and F-actin filaments of C2C12 cells. **A**, **D**, **G** DAPI-stained microcarriers showed C2C12 cell adhesion on day 1 and oval nuclei by day 7 in 1G and µG conditions. **B**, **E**, **H** Phalloidin-stained F-actin filaments showed cell body elongation after 7 days, particularly in µG condition, with less pronounced extension in the 1G bioreactors. **C**, **F**, **I** Merged images of DAPI and F-actin staining illustrated overall cell morphology and cytoskeletal structure. **J** Quantification of nuclei aspect ratio showed increased nuclei elongation in both dynamic conditions, significantly higher in stirred bioreactor. **K** Quantification of cell body length revealed significant elongation in µG condition. *n* = 4 and * indicates significant differences at *p* ≤ 0.05.

To evaluate the effects of µG on cell shape and cytoskeletal organization, we stained C2C12 cells with Phalloidin and DAPI. C2C12 cells adhered firmly to microcarriers on day 1, exhibiting the regular morphology typical of C2C12 cells on T-flask surfaces (Fig. 4A–C). DAPI staining revealed elongated nuclei on the microcarriers, suggesting that cell nuclei underwent stretching over the 7-day culture period. This elongation was more pronounced in dynamic conditions than in static conditions (Fig. 4A, D, G). As described in the “methods” section, the aspect ratio used to quantify nuclei elongation seemed to increase in both 1G and µG conditions (Fig. 4J). The increase in the aspect ratio of nuclei (length/width) for the 1G bioreactor was statistically significant (*p* = 0.0159) compared to day 1 (Fig. 4J). Staining of F-actin filaments in the cell cytoskeleton showed more extensive cell body elongation on the microcarriers after 7 days of culture in both dynamic conditions compared to day 1 (Fig. 4B, E, H). However, the extension was less pronounced in the 1G bioreactor, while cell body extended over longer distances on the microcarriers in µG condition (Fig. 4E, H). Quantitation of these images confirmed that cell body length increased in both µG and 1G conditions, though the increase was higher and statistically significant (*p* = 0.0219) in the µG bioreactor (Fig. 4K). All in all, our results indicate that both dynamic culture conditions promote cell elongation and cytoskeletal reorganization in C2C12 myoblasts.

### 1 G promoted enhanced myogenic differentiation

**Fig. 5:**
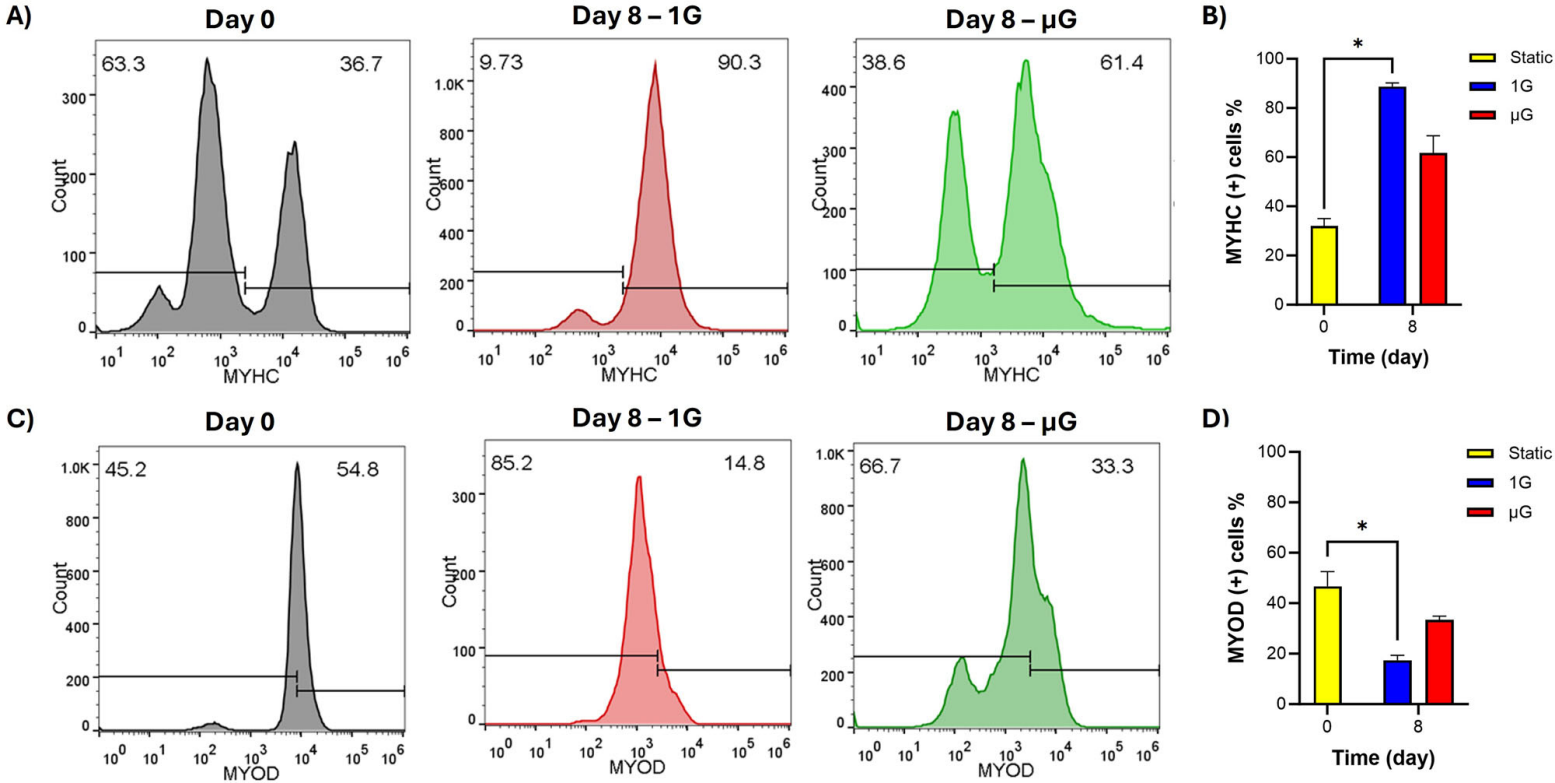
Flow cytometry analysis of myogenic markers of cells cultured in µG and 1 G bioreactors. **A**, **C** Representative flow cytometry images of C2C12 cells at Day 0 and Day 8 in 1G and µG bioreactors. **B**, **D** Quantitative analysis of the percentage of MYHC- and MYOD-positive cells at Day 0 and Day 8 revealed a higher decrease in MYOD-positive cells and a lower increase in MYHC-positive cells in the µG bioreactor compared to the 1G bioreactor. This suggests a delayed transition from the proliferative phase to differentiation in µG, with impaired muscle cell differentiation. *n* = 3 and * indicates significant differences at *p* ≤ 0.05.

To quantify the extent of myogenic differentiation, we analyzed the expression of myogenic markers. The MYHC-positive cell population increased to 90 ± 5% (*p* = 0.0219) in the 1 G bioreactor, compared to 35 ± 7% in T-flasks at day 0, and rose to 60 ± 6.5% in the µG condition (Fig. 5B). Meanwhile, the percentage of MYOD-positive cells in T-flasks at day 0, just before seeding onto the microcarriers, was 54.8 ± 8.5%. After 7 days of culture, this percentage decreased to 33 ± 8% in the µG condition and 15 ± 6.5% (*p* = 0.0219) in the 1G condition (Fig. 5D).

### 1 G advanced muscle differentiation compared to µG, with significant increases in myogenic genes expression

**Fig. 6:**
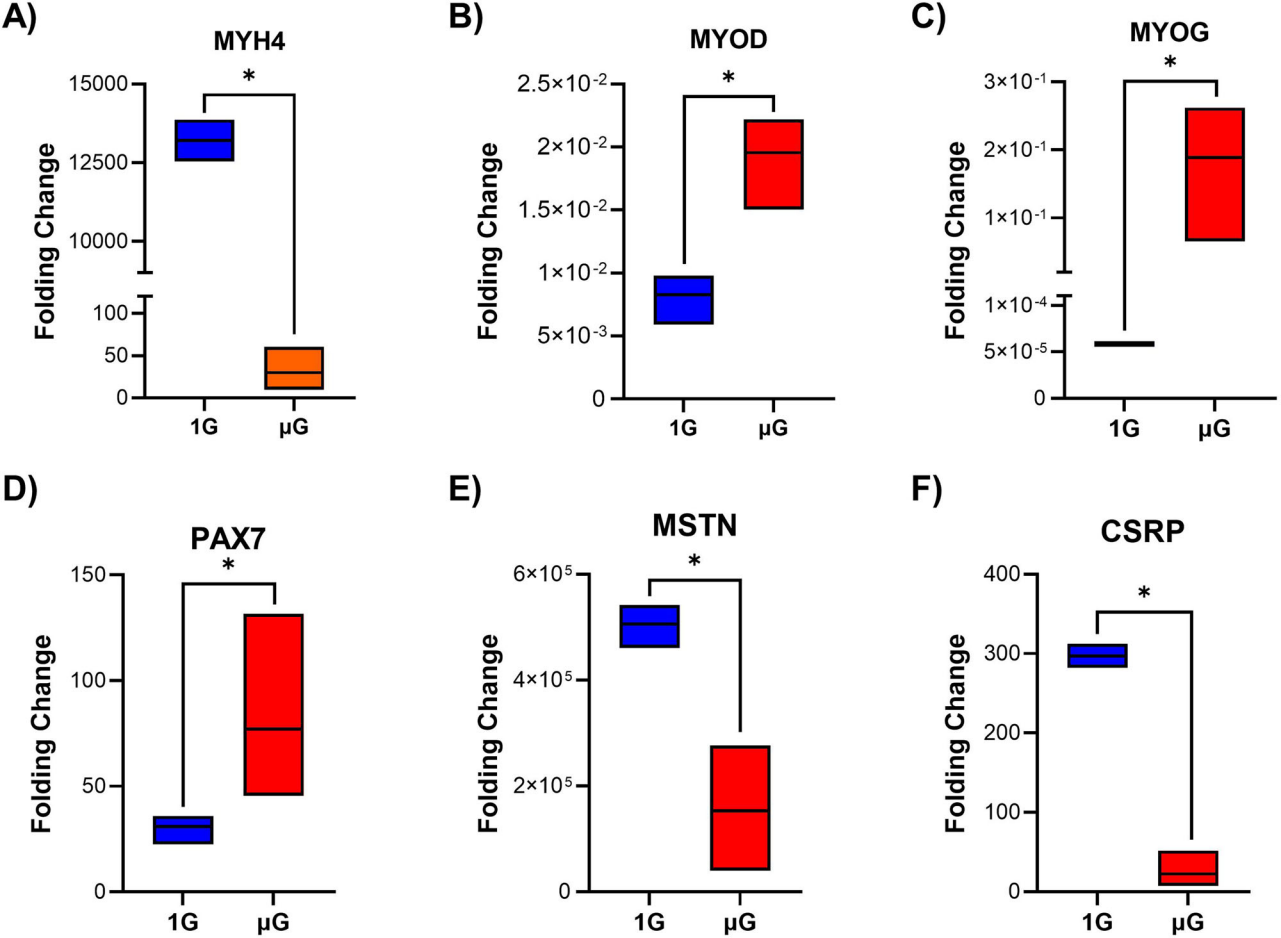
Analysis of myogenesis differentiation-related genes for cells cultured in µG and 1 G bioreactors. **A** MYH4 expression levels showed a significant fold decrease in the µG bioreactor compared to the 1G bioreactor, indicating impaired muscle differentiation in the µG. **B**, **C** MYOD and MYOG gene expression increased in µG bioreactor relative to time zero cells, suggesting an initial commitment to myogenesis, but with a more pronounced decrease in expression observed in the 1G bioreactor, indicating further progression toward differentiation. **D** PAX7 expression levels increased in both the 1G and µG bioreactors, with a greater fold increase in µG samples, reflecting an incomplete transition to mature muscle cells in the µG bioreactor. **E**, **F** MSTN and CSRP expression levels also increased in both conditions, but with a smaller fold increase in the µG samples, suggesting a reduction in muscle growth and extracellular matrix formation in µG. All differences were statistically significant. *n* = 3 and * indicates significant differences at *p* ≤ 0.05.

The fold change in the mRNA expression of myogenesis differentiation-related genes was compared to time zero 2D control samples. The findings from quantitative RT-PCR showed that the fold change of MYH4 increased up to 15,000 ± 2500, compared to 35 ± 5 fold change in µG condition (Fig. 6A). MYH4 is known to be highly expressed in the later stages of muscle cell differentiation^27,28^. MYOD and MYOG, markers of early differentiation^29–31^, dropped by thousands of fold change compared to the mRNA levels in time zero cells, whereas this reduction was less pronounced under µG conditions (Fig. 6B, C). The lower expression levels in 1G, indicates progression towards mature muscle fibers. In contrast, µG shows reduced differentiation suggesting delayed muscle fiber maturation. The expression levels of PAX7, a marker of myoblastic proliferation typically expressed in the early stage of myogenesis^32,33^, increased in both 1G and µG conditions, with a greater fold increase observed in µG samples compared to 1G samples (Fig. 6D). The expression levels of MSTN, a growth factor predominantly expressed in skeletal muscle^34,35^, and CSRP, which is linked to sarcomere formation and muscle growth regulation^36^, increased in both 1G and µG conditions, with a greater fold increase observed in 1G samples compared to µG samples (Fig. 6E, F). This indicates the enhanced muscle maturation under normal gravity. All the changes were statistically significant according to comparisons between two groups per gene, using a *t*-test.

## Discussion

Cultivated meat holds significant potential for space exploration, especially as space agencies plan for long-term habitation on other planets^6,37^. To achieve this, muscle cells, which are adherent, need substrates like microcarriers for growth in specialized bioreactors. To successfully produce meat from animal cells, it is crucial to gain a better understanding of the extent to which these cells can be expanded and differentiated in bioreactors to yield a sufficient quantity and quality of edible tissue.

In this study, gelatin microcarriers were fabricated via a microfluidic flow focusing device that can control the sizes by adjusting the flow rates of gelatin and oil^25,26^. By adjusting the ratio between the oil and dispersed flow rates, we achieved microcarriers in the narrow range of 360 ± 76 μm to 277 ± 35 μm. A higher oil flow rate increases shear forces, leading to smaller droplets, while a higher dispersed flow rate results in larger droplets. This precise control over droplet size is crucial for producing microcarriers with consistent dimensions, ensuring optimal cell growth and differentiation in applications like cultivated meat production.

We initially tested two media types—regular media and differentiation media, which is regular media supplemented with additional growth factor components—under static conditions to determine which would better support C2C12 cell expansion and differentiation. The extra components in the differentiation media play key roles in muscle cell growth and differentiation. While the study does not aim to produce a meat analog free from animal-derived ingredients, the cost of these additional components (e.g., gelatin and serum) was not a primary concern, as the main objective was to look into microcarrier-seeded muscle cells’ differentiation behavior in a simulated µG condition. Horse serum provides essential growth factors and is vital for inducing C2C12 cell differentiation, leading to myotube formation^38^. ITS significantly enhances myoblast differentiation by promoting myotube formation and increasing myogenin expression^39^. EGF, another key component, stimulates cell proliferation and influences myogenic cell activity, as it has been shown to enhance the proliferation of porcine skeletal muscle satellite cells^40^. TGF beta-1, a cytokine involved in various cellular processes, supports myofiber repair and regulates connective tissue formation^41^. Additionally, dexamethasone overexpresses transcription factors essential for myotube formation. Steroid derivatives like dexamethasone are well-documented in triggering differentiation by halting proliferation and inducing the required stress for myotube development^42^. Our preliminary static study demonstrated that by day 7, cells had expanded, spread, and formed bridges across the microcarriers. Differentiation media not only supported greater cell expansion but also significantly increased the number of MYHC-positive cells, leading to its selection for dynamic culture experiments

Although several studies have investigated muscle cell expansion and differentiation in simulated µG^43,44^, our study aimed to extend this research by not only using soft microcarriers made of edible gelatin but also examining the expression of different genes in addition to expansion. This approach allows us to determine the specific stages of muscle cell differentiation under terrestrial 1 G and simulated µG conditions in bioreactors. Furthermore, this study serves as a foundational step for our future research, which will explore different cell types for cultivated meat production, as well as the use of alternative microcarrier types from various sources and structures. In the current study, cell viability decreased in both dynamic conditions, but the 1G bioreactor showed the largest decline, which was also the only statistically significant decrease. This could be attributed to the shear stress generated by agitation in the stirred bioreactors^45^, although viability value still remained high at 87.33%. Despite this, cell expansion in the 1G bioreactor increased approximately threefold over 7 days, indicating that while agitation leads to higher cell mortality, it still supports muscle cell expansion effectively. In comparison, cell expansion was only slightly improved in the µG condition over the same period. Cell detachment from the microcarriers was observed in both bioreactors, though it appeared to occur more frequently in the HARV vessels, leading to the presence of floating cells in the media. Trypan blue staining of these detached cells showed a high proportion of non-viable cells (data not shown). The reduced growth may be associated with cellular apoptosis reported in rotating wall vessels in the literature^46^. We assume that in µG vessels, cells attached to microcarriers are capable of expansion but are more susceptible to detachment. Once detached, these cells often die in the media, resulting in fewer attached cells and creating the impression of inhibited overall cell expansion. This could also explain why the cells that detached enzymatically from the microcarriers were mostly viable, as the viability of the attached cells remained high.

In our study, we observed significant morphological changes in C2C12 cells during dynamic culturing. Quantitative analysis showed an increase in nuclear aspect ratio in both dynamic conditions compared to day 1, with the highest increase observed in the 1G condition. F-actin staining, on the other hand, revealed that the cells exhibited more pronounced elongation in the µG condition. The observed differences in morphological changes under various dynamic conditions can be attributed to the distinct mechanical environments created by these systems. The low shear environment in HARV bioreactors likely creates a more favorable setting for shear-sensitive cells such as muscle cells, allowing cells to spread and elongate more freely^47,48^. In contrast, stirred bioreactors generally expose cells to higher shear forces due to the agitation caused by impellers. While this can enhance nutrient and oxygen transport, excessive shear stress can negatively affect health and function of the cells^47^. The higher shear forces might induce a stress response in the cells, leading to less pronounced elongation as the cells attempt to protect themselves by maintaining a more compact shape.

Flow cytometry was employed to quantitatively analyze the expression of specific proteins including MYHC and MYOD. The observed increase in the MYHC-positive cell population in both the 1G and µG conditions reflects a shift in muscle cell differentiation over the course of the experiment. The substantial rise to 90 ± 5% MYHC-positive cells in the 1G bioreactor suggests that terrestrial gravity strongly support the maturation of muscle cells, likely due to the enhanced availability of mechanical cues that promote myogenesis. In contrast, the lower increase in the µG condition indicates that µG presents a different environment that may challenge or delay the differentiation process. After 7 days of culture, the percentage of MYOD-positive cells decreased in both µG and 1G conditions, with a more pronounced reduction observed in the 1G bioreactor compared to the µG condition. The less pronounced decrease and increase in MYOD-positive and MYHC-positive cells, respectively, under the µG condition implies a delay or a partial arrest in the differentiation process, which may be due to the altered mechanical signaling environment in µG. The µG condition can alter cell signaling pathways and cytoskeletal dynamics, potentially affecting the cells’ ability to fully commit to a myogenic lineage^49,50^. Research has shown that µG affects the structure and function of the cytoskeleton, leading to changes in the transmission of mechanical signals that are essential for myogenic differentiation^51^. In the current study, RT-qPCR analysis of key myogenic markers across various stages of myogenesis, including proliferation/early myogenesis (PAX7, MYOD), mid differentiation (MYOD, MYOG), and late differentiation and maturation (MYH4, CSRP, and MSTN), revealed gene expression differences between the 1G and µG conditions, supporting the flow cytometry results. The significant increase in MYH4, a marker of advanced differentiation, under 1 G aligns with its role in advanced muscle development, as seen in studies where MYH4 was associated with muscle fiber hypertrophy and growth^52,53^. The limited increase in the µG suggests that µG impairs this maturation process, likely due to insufficient mechanical cues. MYOD and MYOG, markers of early and mid-differentiation^29–31^, decreased by thousands of fold change in 1G compared to time zero, indicating advanced differentiation in these cells. In contrast, the reduction in MYOD and MYOG expression under µG conditions was less pronounced, indicating delayed maturation. PAX7 expression was higher under the µG condition compared to 1G. This observation reflects a greater transition from myoblast linage to more mature stages in the 1G^31^. Conversely, higher PAX7 expression under µG could indicate a retention of muscle cells to the earlier stages of differentiation. CSRP, linked to sarcomere formation and muscle growth regulation^36^, and MSTN, a secreted growth factor expressed predominantly in skeletal muscle^34,35^, both increased in 1G and µG conditions, with a higher rise in the 1G condition. This indicates more effective muscle ECM development in the 1G condition, highlighting the enhanced muscle maturation under normal gravity. The CSRP gene serves as a mechanosensor in the development of muscle cells. Its expression is directly influenced by signals from motor and sensory neurons. This gene has a regulatory function, and excessive expression can initiate the process of autophagy. Conversely, insufficient stimulation and expression of this gene can also activate this pathway. The interaction of this gene with LIM protein is essential in controlling this process. Research indicates that the rotation of microcarriers containing muscle cells can activate this protein, speeding up the process of myogenesis and preventing cellular autophagy during differentiation^54^. MSTN is a gene from the TGF-beta family that decreases the growth of myoblast cells. Mutations in this gene are linked to muscle hypertrophy. It helps in the development of myocytes and the production of myotubes by reducing the expansion of myoblast cells during the final stages of myogenesis. This gene plays a crucial role in defining the ultimate function of muscle cells^55^. Given that dexamethasone was used in the differentiation culture medium to accelerate C2C12 cell differentiation, the resulting negative feedback mechanism should lead to reduced expansion, cytoplasmic morphological changes, and cell elongation, while also increasing F-actin elongation. All of these were observed in our final differentiated cells in both 1G and µG conditions.

The flow cytometry and RT-qPCR results show that cells under 1G had advanced to later stages of differentiation. An original and important finding of this study is that the differentiation process is notably delayed and progresses more slowly under the µG condition, particularly at the early stages. The limited expansion and differentiation of muscle cells under µG highlight the need for strategies to enhance myogenesis in space environments. The absence of mechanical stimuli in µG hinders both early and late-stage muscle differentiation, making it challenging to produce mature muscle tissue for cultivated meat. However, the established protocol indicates that although myogenesis is slower in µG, it remains feasible and requires the appropriate chemical and physical inducers to reach sufficient efficiency.

The study’s findings are based on C2C12 murine muscle cells, which limits their applicability. However, the current setup provides a viable platform for studying muscle cell expansion and differentiation in simulated microgravity, and our future research will explore muscle cells from other species to broaden the applicability of these results. Employing targeted growth factors to activate specific myogenesis pathways and using specialized surface coatings on microcarriers could be promising approaches to enhance muscle cell expansion and myogenesis in the µG environments. While the findings offer insights into the challenges of µG, there remains a gap between these experimental results and their practical applications for space-based cultivated meat production. To address this gap, it is essential to investigate how these laboratory-scale findings can be scaled up for larger microgravity bioreactors and production systems in space, particularly through the use of cell-seeded microcarriers during research-based space travels.

This study highlights the potential of HARV bioreactors combined with muscle cell-seeded microcarriers as a platform for studying muscle cell expansion and differentiation in simulated µG environments, with promising implications for cultivated meat production in space. Gelatin microcarriers effectively supported cell adhesion and expansion; however, cell expansion was notably higher in the 1G condition than in the µG. Although flow cytometry and RT-PCR analyses revealed that myogenesis occurred in both 1G and µG, the process was less pronounced under the µG condition. µG condition significantly delayed myogenic differentiation, as evidenced by less MYHC-positive cells and less pronounced reduction of MYOD-positive cells observed in the µG condition compared to the 1G condition. Quantitative RT-PCR analysis of key myogenic markers revealed that proliferation/early myogenesis markers PAX7 and MYOD, as well as mid-differentiation markers MYOD and MYOG, showed higher expression in µG compared to 1G, indicating early-stage differentiation in µG. Conversely, 1G conditions demonstrated higher expression of late differentiation and maturation genes, including MYH4, CSRP, and MSTN, suggesting that muscle cells in 1G had progressed past mid-differentiation stages, with more cells in late differentiation. These results highlight the challenges of achieving complete muscle cell differentiation in µG. Muscle cells under µG were primarily in early differentiation stages, while cells in the 1G bioreactor progressed to later stages, indicating that µG initiates differentiation but at a slower rate. This study’s binary design, examining only 1G and µG conditions, limits a full understanding of gravitational effects. Future investigations should incorporate intermediate gravitational conditions, such as partial gravity or hypergravity, alongside the use of physical inducers and specialized microcarriers with structural mechanical cues to enhance muscle cell expansion and promote myogenesis in microgravity environments.

## Methods

### Fabrication of gelatin microcarriers

A flow-focusing microfluidic device with 600-micrometer channels was designed using Blender 4.2 LTS, which is an open source software. The CAD file was processed and sliced using CHITUBOX Basic V2, with a layer thickness set to 20 µm. Each layer was exposed for 5 seconds at 100% light engine intensity, while the first layer received an extended exposure of 30 seconds. The 3D model of the microfluidic device was then uploaded to a stereolithography 3D printer (Anycubic Photon Mono 4K, Anycubic 3D Printing, China), and printed using a clear 3D printing UV sensitive resin (Anycubic, China). After printing, the device was washed with isopropanol to clean the channels and allowed to dry in preparation for microcarrier fabrication. Gelatin powder from porcine skin (Sigma) was dissolved in phosphate buffer saline (PBS) (Sigma) and placed in a 37 °C incubator to ensure complete dissolution. The gelatin solution was then transferred to a 10 mL syringe and mounted on a syringe pump (Pump 11 Elite from Harvard Apparatus, USA). Meanwhile, 10% Span 80 (Sigma) was dissolved in heavy mineral oil (Fisher Chemical) and loaded into a 50 mL syringe, which was mounted on a separate syringe pump. Polyethylene (PE) tubing was used to connect the syringes to the inlets of the microfluidic device. After investigating different flow rates of oil as described in the next section, the flow rates for the gelatin and oil were set at 10 μL/min and 130 μL/min, respectively, and were used to fabricate the gelatin microcarriers for culturing cells on them. These rates may vary slightly with each batch of microcarrier fabrication. Fabricated gelatin microcarriers were collected in a tube kept in an ice bath to maintain their coolness and integrity. To remove the oil, the microcarriers were centrifuged and washed three times with PBS containing 1% dish soap, followed by three washes with 25% ethanol in PBS, and finally rinsed three times with PBS. All washing steps were performed with cooled solutions. The PBS was then removed as much as possible to form a slurry of microcarriers in the tube. A 16% TI transglutaminase (Moo Gloo, Modernist Pantry, USA) powder was dissolved in PBS and then added to the slurry at a 1:1 ratio (microcarriers slurry: transglutaminase solution). The microcarriers were then heated in a water bath at 45 °C for 1 h for proper enzyme crosslinking. Following this, they were inactivated by being heated at over 80 °C for 10 min. The microcarriers were then washed twice with PBS, centrifuged to remove the PBS, and subsequently 70% ethanol was added. They were stored at room temperature for 1–2 h to sterilize. Subsequently, ethanol was removed, and the microcarriers were washed twice with PBS. Finally, the microcarriers were stored in PBS for subsequent cell culture.

### Microcarrier size characterizations

The size of the fabricated microcarriers was varied by changing oil flow rates while maintaining a constant gelatin flow rate of 10 μL/min. Following the washing steps, 50 μl the microcarrier suspension was placed in a 96-well plate for bright field imaging utilizing a Zeiss Axio Observer microscope (Germany). Subsequently, the average diameter was calculated by measuring the diameter of ~100 microcarriers with ImageJ software (National Institutes of Health, Bethesda, MD, USA). The PDI for each group of microcarriers, corresponding to different oil flow rates, was then evaluated using the following equation (Eq. 1):

1 $${PDI}=\frac{\sigma }{{Dm}}$$

*σ* represents the standard deviation of microcarrier sizes, while Dm indicates the mean diameter of the microcarriers.

### Cell culture on gelatin microcarriers

The murine skeletal muscle cell line C2C12 cells (American Type Culture Collection, Manassas, VA, USA) were cultured in regular complete media, comprising Dulbecco’s Modified Eagle Medium (DMEM) (Gibco) with 10% fetal bovine serum (FBS) (Gibco), 1% penicillin/streptomycin (Gibco), and 1% GlutaMAX (Gibco), at 37 °C in a humidified 5% CO_2_ incubator until they reached 70–80% confluency. Twelve-well plates were prepared for cell seeding on the microcarriers for bioreactor experiments by dissolving 1.5% agarose (Fisher BioReagents) in PBS and coating it on the bottom of the plates. The plates were then sterilized using UV light. Cells from passages less than 5 were used for seeding on the microcarriers. The microcarriers were pre-incubated in a tube with DMEM overnight. The next day, the DMEM media was removed, and the microcarriers were added to the well plates to cover the agarose surface. Cells were trypsinized using 0.05% trypsin-EDTA (Gibco) for 5 min, counted, and 300,000 cells per well were seeded onto the microcarriers. The plates were then incubated at 37 °C with 5% CO_2_ for 2–3 h, shaking every 15–20 min to ensure cell attachment. Following this, media was added to the well plates, which were then incubated overnight before loading into the bioreactors.

### Determining media with enhanced expansion properties

Studies have reported that muscle cells exposed to µG undergo significant physical changes, leading to a loss of muscle mass^27,28^. Additionally, it has been found that µG conditions reduce the expansion capacity of C2C12 cells^43^. To address this issue, we aimed to use cell culture media with enhanced expansion, specifically for meat production purposes. To identify the optimal culture, we conducted a preliminary study using static culture. C2C12 cells were seeded on gelatin microcarriers in the well-plates and cultured under static conditions for up to 7 days. They were split into two groups, with one group in regular media and the other in differentiation media. The regular media was the same as the one used for culturing the cells. The differentiation media consisted of DMEM with 5% FBS, 5% horse serum (Fisher Scientific), 1% penicillin/streptomycin, 1% GlutaMAX, 1% sodium pyruvate (Gibco), 0.5% insulin-transferrin-selenium (Gibco), 50 ng/ml EGF (Stemcell technologies) and 10 ng/mL TGF beta-1 protein (abcam). To enhance differentiation, 5 µg/ml dexamethasone water soluble BioReagent (Sigma), a synthetic glucocorticoid known for reducing expansion and promoting myogenic differentiation by modulating gene expression, was added to the differentiation media on day 5. This timing, 48 h before the culture was completed, allowed sufficient time for the C2C12 cells to transition from an expansion state to a differentiated state, resulting in enhanced myogenesis. Expansion and differentiation of the cells were evaluated at days 1, 3, and 7 to identify optimal media for bioreactor systems, focusing on enhancing both expansion and differentiation. The evaluation methods employed in this study were similar to those used in dynamic conditions and further details on the assessment of cell expansion and differentiation are provided in the following sections.

### µG and 1G dynamic culture in bioreactors

**Fig. 7:**
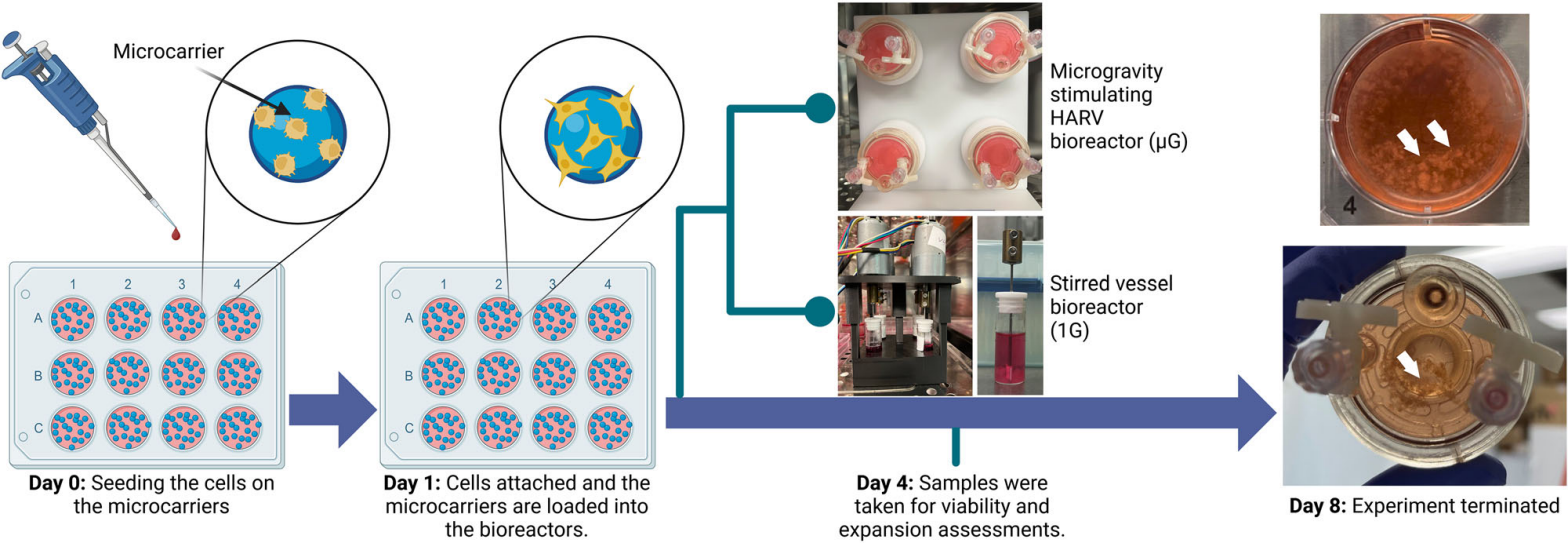
1G and µG bioreactor systems and experimental timeline. Microcarriers were cultured in well plates for 1 day before being transferred into the simulated µG and 1G bioreactors: (1) RCCS-4 with HARVs for a simulated µG environment and (2) custom stirred vessels for standard 1G conditions. Microcarriers were cultured in the bioreactors for 7 days and were analyzed for different assessments on days 4 and 8. White arrows show aggregates of cell-seeded microcarriers produced in both bioreactor systems. Created in BioRender.com.

After 1 day of culturing the microcarriers in well plates, they were transferred into the bioreactors. Two types of dynamic bioreactor cultures were utilized to investigate the impact of simulated µG and terrestrial gravity (1G) conditions on cells expansion and differentiation (Fig. 7). One condition was the use of a rotary cell culture system (RCCS-4; Synthecon, Inc., TX, USA), developed by NASA researchers, that comprised of four High Aspect Ratio Vessels (HARVs)^56^. HARV bioreactors are referred as RWVs, establish a low-shear stress environment that enhances suspended particle motion and simulates µG conditions with a dynamic gravitational vector^57,58^. The HARV system consisted of four vessels, each with a 10 ml volume. The second condition utilized custom-developed stirred vessels designed in the laboratory to replicate the 1G conditions as a terrestrial control for µG condition. This system included 4 vessels, each with 7 ml capacity. Microcarriers were loaded into the bioreactor vessels, with 0.75 ml of microcarriers for the HARV conditions and 0.5 ml for the stirred vessels. The vessels were then filled with differentiation media, and the bioreactors were operated inside a 37 °C humidified incubator with 5% CO_2_ for a duration of 7 days. Each day, half of the media was replaced with fresh media.

### Cell viability and expansion analyses

A 100 μL sample of microcarrier suspension was collected after one, three, and 7 days of culture in both static and dynamic bioreactor conditions. Manual agitation was used to ensure uniform sampling from 12-well plates (for static culture) and bioreactor vessels (for dynamic culture). The number of microcarriers was counted after photographing them using a light microscope. The media was discarded, and the microcarriers were washed twice with PBS. Cells were then detached from the microcarriers by using 0.5% trypsin-EDTA and incubating for 5–10 min at 37 °C in a humidified 5% CO_2_ incubator. The viability and expansion of the C2C12 cells were quantified using the trypan blue exclusion assay. The detached cells were mixed with trypan blue dye in a 1:1 ratio, and the live and dead cells were counted using a hemocytometer. The viability and the number the cells per microcarrier were calculated using the following equations (Eqs. 2 and 3):

2 $${\rm{Viability}}\left( \% \right)=\frac{{\rm{Number\; of\; live\; cells}}}{{\rm{Total\; number\; of\; cells}}}\times 100$$

3 $${\rm{Number\; of\; cells\; per\; microcarrier}}=\frac{{\rm{Number\; of\; live\; cells}}}{{\rm{Number\; of\; microcarriers}}}$$

The viability of the cells on the microcarriers was also evaluated qualitatively using fluorescence imaging with a calcein/ethidium assay (Invitrogen). Calcein, which penetrates live cell membranes, produces a green fluorescence signal. Conversely, ethidium penetrates only the damaged membranes of dead cells, emitting a red fluorescence signal. The sampled microcarriers were washed twice with PBS and then incubated with a PBS solution containing 4 μM calcein AM and 2 μM ethidium homodimer-1 for 1 h at room temperature, shielded from light. After staining, the microcarriers were washed twice with PBS, visualized using a Zeiss AXIO fluorescent microscopy (Germany), and imaged for analysis.

### Immunofluorescence staining of C2C12 cells on microcarriers

To observe cell shape and morphological changes, cells were stained using immunofluorescence staining for visualizing the F-actin filaments of the cells. Microcarriers were washed twice with PBS and fixed in 4% paraformaldehyde (VWR) at 4 °C overnight. The next day, the microcarriers were washed with PBS and then blocked with a 5% w/v bovine serum albumin (VWR) solution in 0.3% Triton X-100 (BIO BASIC) dissolved in PBS while being incubated at 4 °C overnight. The following day, the BSA solution was discarded, and the samples were incubated overnight at 4 °C with 4 µg/mL of Alexa Fluor™ 488 Phalloidin (Thermo Fisher Scientific) in 5 times diluted blocking solution. Following removal of the antibody solution, the cell nuclei were stained with a DAPI solution (4’,6-Diamidino-2-Phenylindole, Dihydrochloride, Thermo Fisher Scientific) in PBS and then incubated for 30 min at room temperature in the dark. After two washes with PBS, the microcarriers were placed on a glass slide and examined under the fluorescence microscope to observe cell morphology and capture images. In the differentiation process from myoblasts to myotubes, cellular morphology changes from a round shape to an elongated tubular shape as the cells elongate, adhere, and fuse into multinucleated myotubes^59,60^. Thus, the images were processed using ImageJ software to quantitate morphological changes. Nuclei elongation was measured by determining the nuclear aspect ratio, defined as the ratio of the longest to the shortest diameter of the nuclei. Using ImageJ software, nuclei from several images in each dynamic group were randomly selected and analyzed. Furthermore, F-actin-stained fibers were additionally analyzed to quantify cell body length indicating potential morphological changes linked to cellular differentiation. Using ImageJ software, the lengths of the fibers were manually measured from randomly selected cells.

### Flow cytometry

Flow cytometry was employed to analyze myogenic markers, including MYHC and MYOD, because it enables precise measurement of protein expression in individual cells. This method allows for the assessment of the proportion of cells expressing the myogenic markers and their relative abundance within the cell population. MYOD is a key transcription factor involved mostly in the early stages of myogenesis, serving as an early factor that signals the commitment of cells to the muscle lineage and drives the expansion of myogenic-directed cells^61^. MYHC, on the other hand, is a later-stage marker that indicates the maturation and functional development of muscle fibers^62^. Cells were detached from microcarriers using a trypsin-EDTA 0.5% solution after washing with PBS. The microcarriers were separated from cells using a 0.07 mm cell strainer. 5000 cells/µl were stained using PE-conjugated anti-mice MYHC antibody (also known as MF20, BD-USA) and FITC-conjugated anti-mice MyoD antibody in PBS containing 2% bovine serum albumin (BSA). Cells were diluted in 500 µl PBS after 30 min of incubation and read through a flow cytometry machine (Attune NXT-Thermo Fisher- USA).

### RNA extraction, cDNA synthesis, and real-time polymerase chain reaction (RT-PCR)

To gain a deeper understanding of myogenic differentiation, we performed RT-PCR analysis on six myogenic genes. This technique allowed us to examine gene expression patterns, complementing the protein-level data obtained from flow cytometry. By studying gene expression, we could determine the number of cells undergoing myogenic differentiation and their stage within the differentiation process.

The total RNA of all samples was extracted using TRIZOL reagent (Invitrogen-USA). The pellet of cells after each condition was resuspended in TRIZOL until the TRIZOL solution was clear. All samples were stored at −80 °C, and after the completion of all experiments, the total RNA of all samples was extracted simultaneously based on TRIZOL-based RNA extraction protocols. 100–2000 ng of total RNA were reverse-transcribed to single-stranded DNA using a cDNA synthesis kit (ABM-Canada). Specific primers for MYH4, MYOD, MYOG, PAX7, CSRP, and MSTN were designed using GeneBank primer designing tools (Table 1). Quantitative RT-PCR was performed using SYBR Green Mastermix to evaluate the relative gene expression of the mentioned genes, comparing all samples to time zero non-differentiated C2C12 cells.

**Table 1:** Primer sequences used for RT-PCR

Genes	Forward sequence	Reverse sequence
GAPDH	TTGTCAGCAATGCATCCTGC	CCATCCACAGTCTTCTGGGT
PAX7	AGTTCGATTAGCCGAGTGCT	ATGCCGTGGTGAGCCATCT
MyoG	CCCAACCCAGGAGATCATTTGC	CAGTTGGGCATGGTTTCGTC
MyoD	CTGCTCTGATGGCATGATGGAT	ACTATGCTGGACAGGCAGTC
MYH4	TGGCCACAGACACTGCTGTT	TCAGCAACTTCGGTGCCGTCT
MSTN	GGAGAAGATGGGCTGAATCC	TGGGTGCGATAATCCAGTCC
CSRP	GCATGGCCTGCAGGAAAGCTCT	TGTCTGTGCTGAGGCAGCCA

### Statistical analysis

Statistical analyses were performed with GraphPad Prism software package version 9.4.1. As all groups had small sample sizes, non-parametric statistical Kruskal–Wallis one-way analysis of variance (ANOVA) followed by Dunn’s multiple comparisons test was used. For RT-PCR analyses involving comparisons between two groups per gene, a *t*-test was applied. The figures depict the median with standard deviation, and statistical significance was accepted for *p* values of less than 0.05.

## Data Availability

The authors confirm that all data supporting this study’s findings are included in the paper.
